# Characterizing the Shearing Stresses within the CDC Biofilm Reactor Using Computational Fluid Dynamics

**DOI:** 10.3390/microorganisms9081709

**Published:** 2021-08-11

**Authors:** Erick Johnson, Theodore Petersen, Darla M. Goeres

**Affiliations:** 1 Department Mechanical Engineering, Montana State University, Bozeman, MT 59717, USA; erick.johnson@montana.edu (E.J.); theo.m.petersen@gmail.com (T.P.); 2 Center for Biofilm Engineering, Montana State University, Bozeman, MT 59717, USA

**Keywords:** CDC biofilm reactor, shear stress, computational fluid dynamics

## Abstract

Shearing stresses are known to be a critical factor impacting the growth and physiology of biofilms, but the underlying fluid dynamics within biofilm reactors are rarely well characterized and not always considered when a researcher decides which biofilm reactor to use. The CDC biofilm reactor is referenced in validated Standard Test Methods and US EPA guidance documents. The driving fluid dynamics within the CDC biofilm reactor were investigated using computational fluid dynamics. An unsteady, three-dimensional model of the CDC reactor was simulated at a rotation rate of 125 RPM. The reactor showed turbulent structures, with shear stresses averaging near 0.365 ± 0.074 Pa across all 24 coupons. The pressure variation on the coupon surfaces was found to be larger, with a continuous 2–3 Pa amplitude, coinciding with the baffle passage. Computational fluid dynamics was shown to be a powerful tool for defining key fluid dynamic parameters at a high fidelity within the CDC biofilm reactor. The consistency of the shear stresses and pressures and the unsteadiness of the flow within the CDC reactor may help explain its reproducibility in laboratory studies. The computational model will enable researchers to make an informed decision whether the fluid dynamics present in the CDC biofilm reactor are appropriate for their research.

## 1. Introduction

Biofilms are self-organized communities of bacteria often associated with surfaces and aqueous environments encased in an extracellular polymeric substance [1]. Biofilm growth, as with all living systems, is responsive to multiple factors including temperature, availability of carbon and trace elements, the oxygen concentration, and the composition of the microbial community. As a biofilm is often associated with surfaces, the fluid dynamics present where the biofilm is growing are important to consider. In the laboratory, biofilm reactors are engineered to represent the spectrum of environments where biofilms thrive. Intuitively, a biofilm growing as a bacterial matt in a hot pot located in Yellowstone National Park, US [2], will have different defining characteristics than a biofilm growing on a monument in the Mediterranean [3], or on an infected indwelling medical device [4]. If the research goal is for laboratory data to be predictive of the environment under investigation, then careful consideration is necessary when deciding which biofilm reactor to use. Defining the fluid dynamics present in the environment under investigation and then modeling those fluid dynamics in the laboratory is a useful strategy when deciding which laboratory reactor is most appropriate. In this paper, a computational fluid dynamics model was used to describe the fluid dynamics present in the United States Centers for Disease Control and Prevention (CDC) biofilm reactor.

Multiple laboratory reactors are available for growing biofilms under various conditions [5]. While parameters such as the temperature, carbon source, and composition of microbes may be similar across different protocols that rely on various reactors, the fluid dynamics present are a defining feature unique to a particular reactor, or class of reactors, and are often the defining feature that enables a researcher to decide upon the best reactor to use [6]. Fluid dynamics influence the cell density within a clump, and the stability of the biofilm, with biofilms gown under high shear being more stable than biofilms grown under low shear [7]. Stoodley et al. demonstrated that biofilms grown under higher shear are more strongly adhered and have stronger EPS [8], which explains the increased stability. Fluid dynamics influence the biofilm architecture and the movement of detached clumps over a surface [9]. Practically, these differences in fluid dynamics will influence how the biofilm responds to biocides [10,11,12].

The CDC biofilm reactor was originally designed to study legionella biofilms in potable water [13]. US CDC researchers designed the reactor using the same coupons that were used in the rotating disk reactor [14]. Functionally, the CDC reactor improved upon the design limitations of the rotating disk reactor by including more coupons, placing the coupons in rods that could be sampled over time without disrupting the other samples, and improving the mixing. In 2005, an optimized protocol based on the CDC reactor was published [15], and then in 2007, Standard Test Method E2562 based on the optimized protocol and reactor design was approved by ASTM International [16]. In 2018, Standard Practice E3161 that describes how to grow a biofilm that is used for efficacy testing was approved by ASTM [17]. The CDC biofilm reactor practice E3161 is refenced in the US EPA Guidelines, defining the pathway companies must follow to verify a “kills biofilm” product claim [18].

The CDC biofilm reactor is a continuously stirred tank reactor, where nutrients are introduced at a constant rate and by-products exit at the same rate, meaning there is no net accumulation of liquid. The biofilm is grown on coupons placed in rods suspended from a lid that rests on top of a 1 L beaker with an effluent spout. A baffle centered in the middle of the beaker rotating at a specified speed sets the fluid dynamics present in the reactor. A six-laboratory collaborative study demonstrated that the viable cell log density for a *Pseudomonas aeruginosa* biofilm grown in the CDC biofilm reactor is highly reproducible (SD = 0.2442), with 78% of the variability attributed to among-laboratory sources and 22% of the variability attributed to within-laboratory sources, including within-experiment sources, when sampled following the Single Tube Method [19]. While standardized for growing *Pseudomonas aeruginosa* and *Staphylococcus aureus* biofilms and commonly used for biocide efficacy testing, the CDC biofilm reactor has been used for a range of other applications, proving itself a useful tool for the biofilm research community.

Computational fluid dynamics (CFD) models are powerful tools for defining key fluid dynamic parameters at a high fidelity. This approach has been introduced as a viable tool for other biofilm reactors, including a six-well tissue culture plate [20], the MBEC^TM^ device [21], and a flat, fixed-bed reactor [22]. Both the two- and three-dimensional CFD models showed a higher level of accuracy to experimentally measured dissolved oxygen as compared to a discretization of the transport equations in MATLAB or AQUASIM, though no shear stress values were reported [22]. In models completed for the two rotational reactors, Azevedo et al. [20] reported an average shear stress of 0.317 Pa at 120 RPM, while [21] evaluated the shear stress at a single location with a peak near 1 Pa and an average of approximately 0.66 Pa, with a rotational rate of 150 RPM. Further, a separate focus for research is understanding biofilm growth, clogging, and detachment within porous media [23,24,25]. Modeling studies at the pore scale often rely on coupling multiple resolutions of the Navier–Stokes equations to account for the bulk fluid flow and the flow within the biofilm or media with an even smaller pore geometry. While these approaches are advancing our understanding of biofilms, they have not currently demonstrated the ability to capture the wall shear stress accurately. Only one reference has demonstrated a CFD model for the CDC biofilm reactor, where a 10% hexadecane water mixture spinning at 170 RPM resulted in shear stresses between 0.35 and 0.39 Pa on the inner coupons [26]. That study, however, did not provide sufficient modeling details or descriptions of the fluid dynamics within the system. The purpose of the current study was to use a computational fluid dynamics model to more fully characterize the fluid dynamics present in the CDC reactor.

## 2. Materials and Methods

### 2.1. Computer-Aided Design Geometry

**Figure 1:**
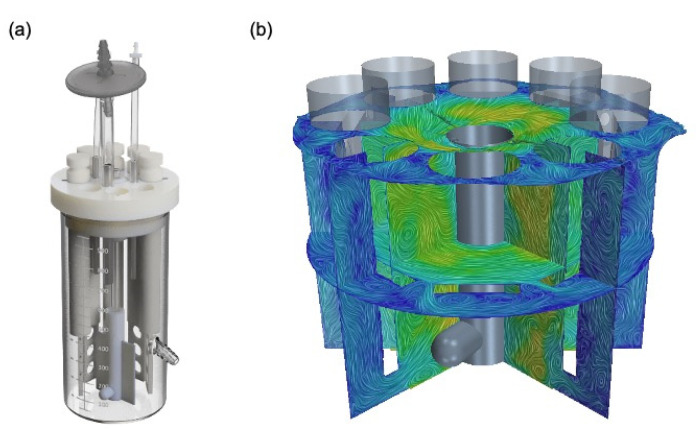
The CDC biofilm reactor (**a**) with the CAD representation of the fluid geometry (**b**) and showing the liquid phase velocity on four cross-section planes at approximately 15.5 s into the simulation.

The three-dimensional computer-aided design (CAD) geometry of the CDC reactor was provided and imported into the CFD software, Siemens Simcenter STAR-CCM+ (Siemens PLM Software, Plano, TX, USA). Using the CAD features within STAR-CCM+, a new three-dimensional geometry was created representing the space occupied by the nutrient bath and air within the reactor, i.e., all the solid materials of the reactor were removed, leaving voids in their stead. The reactor and CAD can be seen in Figure 1. As the air portion is not of direct interest, this geometry was shortened to approximately 10 mm above the effluent spout, allowing room to represent the free surface while also reducing the computational time required to solve the model. Further, to simulate the rotation of the baffle, this new fluid geometry was split into two regions. The first region remains stationary and includes the coupons and their supporting rods. The second region is a rotating cylinder, with a radius of 25 mm between the baffle and coupon surfaces, and encompasses the stir plate magnet and attached baffle.

The surfaces of the coupons were assumed to be flush with their supporting rods. Small offsets in this height will reduce the shear stresses on the edges of the coupons but are unlikely to change the shear stresses over the majority of the coupon surface.

### 2.2. Numerical Methods

STAR-CCM+ (v15.04.008) solves the unsteady, three-dimensional, Reynolds-averaged Navier–Stokes (RANS) equations with the finite volume method throughout a discretization (mesh) of the model geometry. The model was assumed isothermal (25 °C) and incompressible, requiring only a coupling between the continuity and conservation of momentum equations. This coupling uses the SIMPLE algorithm, a first-order temporal scheme. A volume-of-fluid (VOF) approach was used to simultaneously simulate the nutrient bath and air phases, where a percent volume fraction of each fluid was convected and stored in every mesh element [27]. This allowed the free surface to dynamically move and balance the pressures caused by the liquid phase interacting with the solid boundaries of the coupon rods, baffle, and reactor walls. As a side note, a separate simulation was performed where the entire reactor was modeled as a single liquid phase and showed some, though an insignificant amount of, difference compared to the presented values; only the two-phase VOF model is presented here. Due to the significant flow separation around the baffle and coupon rod edges, the shear stress transport (SST) k–w turbulence model was used to close the Reynolds stress tensor and best capture the shear stresses on the coupon surfaces [28]. Gravity is included.

### 2.3. Model and Boundary Conditions

The CDC biofilm reactor was modeled as a combination of a stationary geometry (containing the coupons and coupon rods) and a rotating geometry (containing the central baffle plate and stir plate magnet), where an interface allowing mass and momentum to move between the geometries was updated during every time-step. The rotating geometry spins around its central axis at 125 RPM. A time-step of 0.001 s was determined to provide a suitable level of accuracy and resulted in 0.75° of rotation per time-step. Since the biofilm and nutrients make up less than 1% of the liquid mixture, the simulation assumes that the physical properties of the liquid phase can be approximated as room temperature water. The properties of the air phase were approximated at sea level and were not expected to have an influence on the results.

The initial conditions for this transient model assume the liquid and air phases were at rest, with an initial water height 5 mm above the lower lip of the effluent spout. Since no actual biofilms were grown in the simulation, no new liquid entered the system through the top boundary, which is considered as a pressure outlet allowing air to freely enter and leave through this surface. The liquid and air phases could drain out of the spout to achieve an equilibrium height and were also considered as a pressure outlet. At the first time-step, the baffle began spinning at 125 RPM, with no ramp-up period. The simulation was solved to 33.6 s (70 full revolutions), well past the apparent convergence to a quasi-steady state.

### 2.4. Meshing

**Figure 2:**
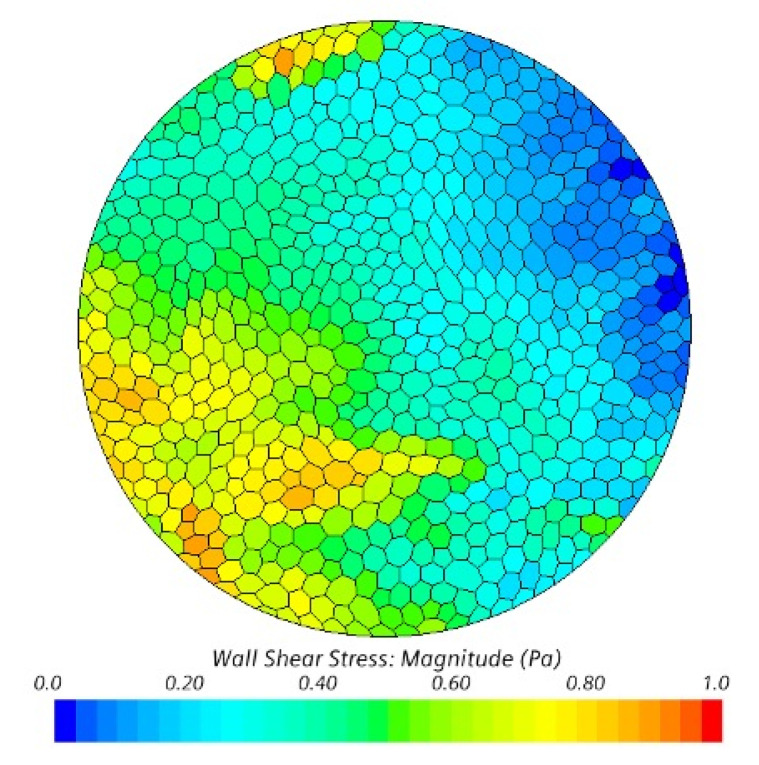
Example of the polygonal mesh on one of the coupon surfaces, colored by the wall shear stress from an arbitrary snapshot in time. Baffle motion and the average fluid flow are from left to right.

A mesh is the discretization of the geometry into smaller elements used to solve a set of governing equations, where their size and shape contribute to the solution accuracy. STAR-CCM+ has a built-in mesh generator that can produce a mesh with n-sided polygons, significantly reducing the number of elements required to obtain results with a similar level of accuracy to a mesh using tetrahedral elements. This has a net effect of reducing the total computational time required to simulate a problem. The entire model had a total of 1,315,700 polygonal elements for the two geometries, with an average element length of 1.3 mm. As the coupon surfaces are of most interest, element sizes were reduced and had an average length of 0.452 mm, with approximately 667 elements on every coupon surface. An example of a coupon surface mesh can be seen in Figure 2. As two regions were modeled, the elements on adjacent surfaces were specified as having the same average size to minimize numerical diffusion across the boundaries.

Additionally, to best capture the fluid boundary layer on the coupon surfaces, and therefore the resultant wall shear stresses, the SST k–w turbulence model was used. The SST modification was introduced to improve upon the sensitivity of the classic two-equation k–w turbulence model [28,29]. Unlike many turbulence models that approximate the boundary layer as a logarithmic profile within the thickness of one element, the k–w and SST k–w models explicitly resolve the velocity at every element and are widely used through a range of applications where a more accurate evaluation of the wall shear stress is necessary [30]. To achieve this level of resolution, these models require highly anisotropic (thin in the perpendicular direction) elements that have faces orthogonal to a surface. A prism layer technique was used on the coupon and coupon rod surfaces, producing these anisotropic elements. A total of 7 prism layers were used, with a growth rate of 1.15 increasing the element thickness away from the surface and smoothly transitioning to the larger polygonal mesh. The *wall y+* is a non-dimensional metric used to determine the suitability of a mesh when capturing the velocity boundary layer in a turbulent model. The SST k–w model requires that the wall *y*+ is <1, with the presented mesh having an average value of 0.692 and a maximum of 1.374. This was sufficient for the presented simulation. The remaining surfaces use a logarithmic approximation to the boundary layer velocity profile, which is standard practice.

A mesh independence study was performed to determine suitable element sizes.

### 2.5. Data Collection and Processing

Pressure and the vector components of shear stress were collected from each coupon surface element every 0.005 s and saved as CSV (comma-separated variables) files. These data were then imported into MATLAB for further data analysis. Due to gravity, hydrostatic pressure increases with fluid depth (to approximately 600 Pa at the bottom of the reactor) and was superimposed on top of the static and dynamic pressures produced from the baffle motion. The total pressure presented here removes the hydrostatic component by subtracting its contribution using the average height of the free surface at each time-step. MATLAB was used to extract the minimum and maximum pressures and shear stresses on each coupon for every time-step. Average values for coupons were weighted by the surface area of the element where the data were collected. Unless otherwise noted, the results were phase locked, meaning that the data from each coupon were temporally shifted by multiples of 45° such that the start of a revolution is defined as the baffle being centered over the coupon center. Data are presented temporally for each coupon, as phase-locked averages of all coupons, and as quasi-steady state averages. Due to the deterministic nature of fluid modeling, the independence of data is not guaranteed between time-steps, and a Lilliefors test fails normality for both the wall shear stresses and pressures. Statistical significance was determined using the non-parametric Kruskal–Wallis H-test as implemented within MATLAB.

### 2.6. Bioflm Growth in the CDC Biofilm Reactor

A *Pseudomonas aeruginosa* ATCC 15442 biofilm was grown on borosilicate glass coupons in the CDC biofilm reactor according to ASTM Method E2562-17. In summary, a colony collected from a streak plate grown on R2A agar (Fisher Scientific, Waltham, MA USA) was added to 100 mL of 300 mg/L Tryptic Soy Broth (TSB) (Fisher Scientific) and grown for 24 h at 36 °C in an environmental shaker. At the end of the 24 h, the viable cell density was approximately 10^8^ CFU/mL and was checked by serial dilution and plating. The CDC biofilm reactor was sterilized and then filled with 500 mL of sterile 300 mg/L TSB. An amount of 1 mL of the inoculum was added to the reactor, and the baffle was set to rotate at 125 RPM. The biofilm grew for 24 h at room temperature (21 ± 2 °C). At 24 h, 20 L of sterile 100 mg/L TSB was connected to the reactor’s influent via a peristaltic pump set to flow at 11.7 mL/min. The effluent line was unclamped, and the biofilm grew for another 24 h with a continuous flow of nutrients, with the baffle rotating at 125 RPM. After a total of 48 h, the pump and baffle were turned off, a rod was removed and gently rinsed in sterile buffered water to remove loosely attached cells, and three coupons from the top, middle, and bottom rod positions were collected for imaging, paying special attention to which side of the coupon faced the baffle.

### 2.7. Imaging the Mature Biofilm

Microscopic imaging was performed on an upright Leica TCS-SP5 Confocal Scanning Laser Microscope using the 488 and 561 nm laser excitation lines. Biofilms were stained with LIVE/DEAD BacLight Bacterial Viability Kit stain (Invitrogen #L7012, Carlsbad, CA, USA) for 20 min, rinsed, and then imaged in a fully hydrated state using extra-long working distance water immersion objectives. The images were processed using Imaris x64 9.2.0 software (Bitplane Scientific Software, Zurich, Switzerland).

## 3. Results

### 3.1. Simulated Results

**Figure 3:**
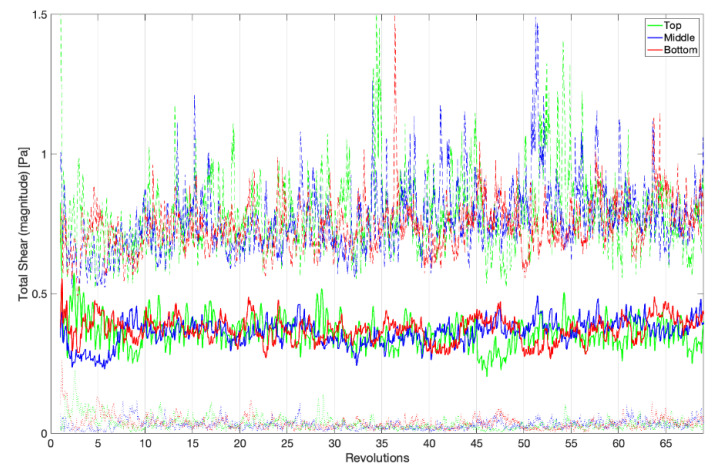
Averages of the total shear stress for the top, middle, and bottom rows of coupons as a function of time. The coupon averages (bold), maximums (dashed), and minimums (dotted) are shown. The average shear stress is area weighted by the element area.

**Figure 4:**
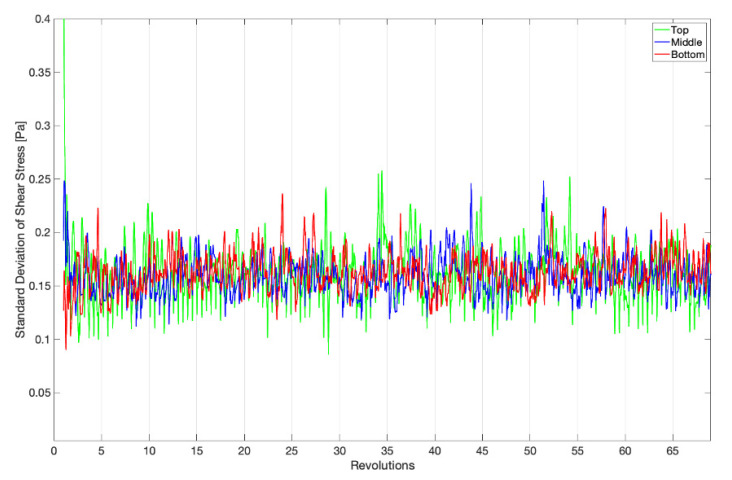
Averages of the instantaneous standard deviations of the total shear stress on each coupon surface for the top, middle, and bottom rows of coupons.

In fluid mechanics, flows may broadly be considered as being steady (time invariant), periodically steady (repeated patterns with a constant frequency), quasi-steady (strong repeatability with some fluctuations), or turbulent. Separately, there is a period of time when transitioning between constant operating conditions, e.g., starting to rotate from a stopped condition, where the observed fluid dynamics are not indicative of the driving response in either state. As it can be seen in Figure 3 and Figure 4, between 5 and 15 revolutions are required for the startup effects to diminish and the flow within the reactor to take on its operational response, which is transitional between quasi-steady and turbulent. All remaining results and plots will omit this startup period and only consider data collected beginning at revolution 20. After the startup period, the standard deviation of the total wall shear stress asymptotes to an average of 0.162 ± 0.023 Pa for the top, middle, and bottom rows of coupons, which can be seen in Figure 4.

#### 3.1.1. Shear Stress

**Figure 5:**
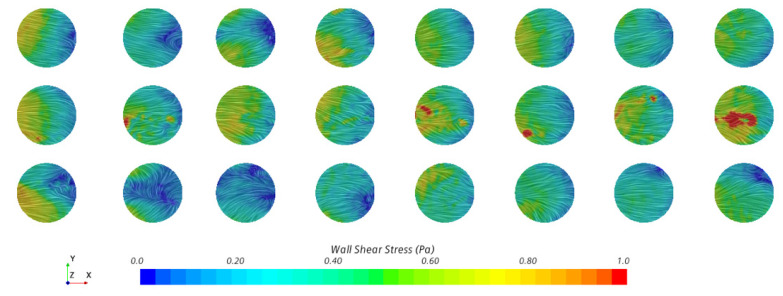
The wall shear stress on all 24 coupon surfaces at 25 s. Every fourth column is opposite each other in the reactor, and the baffle moves from left to right across the coupon surfaces.

The wall shear stress is the product of the shearing rate of the fluid at a surface and a fluid’s dynamic viscosity. As all eight coupons at the same depth within the reactor are anticipated to experience similar flow patterns, the results are differentiated by their row placement within the sample rods, i.e., top, middle, and bottom. Figure 3 shows the row average of the average, maximum, and minimum magnitudes of the shear stress on each coupon surface. Over the final 50 revolutions, the top, middle, and bottom rows experience an average total shear stress of 0.358, 0.368, and 0.368 Pa, respectively. Table 1 lists the average, maximum, and minimum shear stress magnitudes observed between revolutions 20 and 70. Over the final 20 revolutions, these are 0.364 Pa (*p* = 0.130), 0.389 Pa (*p* = 0.0175), and 0.369 Pa (*p* = 0.326), respectively, when compared to 50 revolutions. These small shifts in the magnitude of shear stress are attributed to the low-frequency fluid structures that exist in the system rather than a drift in the average shear stress. As it can be seen, while there are both high- and low-frequency variations in the shear stress on these surfaces, no row appears to show a meaningful difference based upon its location, with *p* = 0.187, and this agrees with the results from [26]. Additionally, comparing the instantaneous standard deviation across the coupon surfaces in Figure 4 to the data in Table 1, the extrema observed in Figure 3 may be important to consider in the analysis of the reactor but do not introduce significant variations across coupon surfaces. An instantaneous snapshot of the wall shear stress on all 24 coupons is shown in Figure 5 and showcases the unsteadiness within the reactor and its effect on the shear stresses each coupon experiences.

**Figure 6:**
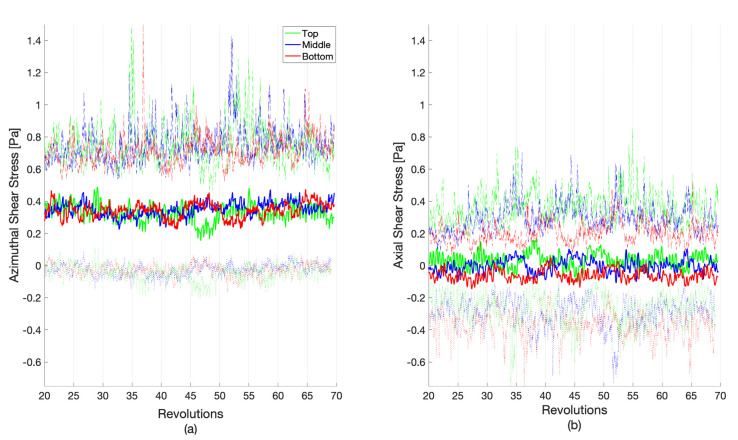
The (**a**) azimuthal and (**b**) axial vector components of the row-averaged shear stress between revolutions 20 and 70. The coupon averages (bold), maximums (dashed), and minimums (dotted) are shown. The average shear stress is area weighted by the element area.

**Figure 7:**
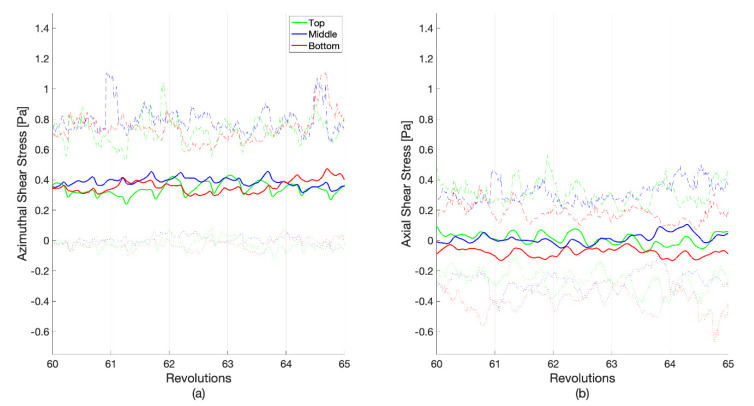
A zoomed-in portion of Figure 6 between revolutions 60 and 65, where the (**a**) azimuthal and (**b**) axial vector components are presented. The coupon averages (bold), maximums (dashed), and minimums (dotted) are shown. The average shear stress is area weighted by the element area.

Shear stress is a vector with components in the reactor that are tangent to the rotating axis (azimuthal) and parallel to the rotating axis (axial). Figure 6 shows the decomposition of the total shear stress; a zoomed-in view of these components between revolutions 60 and 65 is shown in Figure 7. Most of the total shear stress is a result of the azimuthal motion created by the rotating baffle, with a nearly zero pascal average from any vertical movement of the fluid. The average shear stresses in the axial direction are 0.033 ± 0.072, −0.002 ± 0.067, and −0.061 ± 0.067 Pa, for the top, middle, and bottom rows, respectively. A small portion of the azimuthal shear does reverse, as it can be seen in the negative minimum values. This implies that a small portion of the coupon surfaces experiences a flow that is moving slowly in the direction opposite to the baffle. However, more interesting are the large magnitudes of the maximum and minimum shear stresses in the axial direction. While the average axial shear stress is nearly zero, the maximum and minimum values can exceed the average total shear stress in both an upward and a downward direction. This implies a significant amount of mixing within the reactor outside of the rotation plane, with strong, multi-dimensional flow structures. Figure 7 also highlights the different temporal scales in the flow structures. With the single baffle cutting the reactor in half, a twice-per-revolution structure would be anticipated to dominate. While this pulse is generally observed, other flow structures exist at both higher and lower frequencies, indicating turbulent mixing.

**Figure 8:**
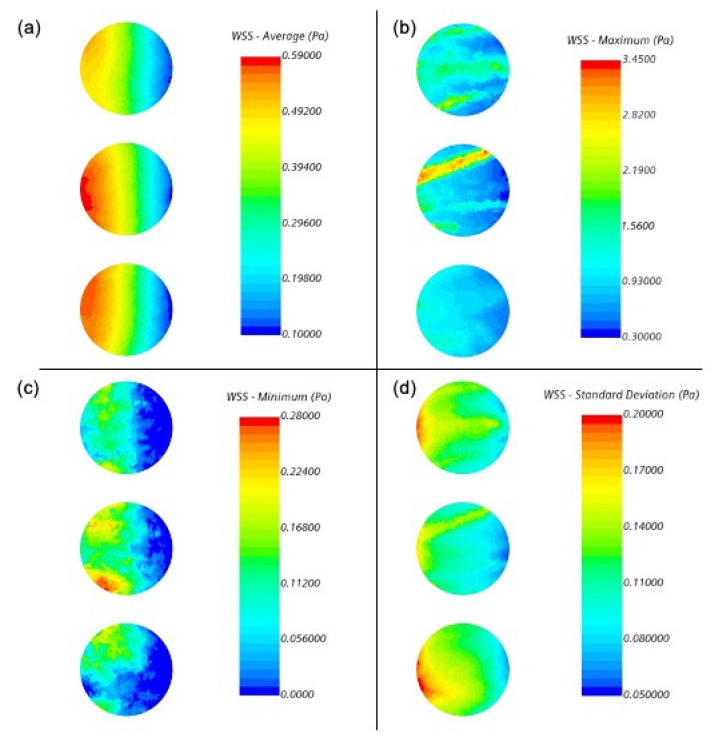
The wall shear stress on one vertical rod of coupons between revolutions 20 and 70, with the baffle moving across the surfaces from left to right. (**a**) is the average wall shear stress magnitude over all revolutions, while (**b**) is the absolute maximum observed during the 50 revolutions, (**c**) is the absolute minimum, and (**d**) is the standard deviation.

An alternative way to consider the shear stress on the coupons is to investigate how it varies across a coupon surface. The time-averaged, maximum, and minimum shear stresses for a single rod at all three vertical locations are highlighted in Figure 8. The baffle moves across the coupon surfaces from left to right, which demonstrates a bias in the average shear in the azimuthal direction. However, the standard deviations observed in Figure 8d demonstrate that nearly the entirety of the coupon surfaces experiences significant fluctuations. The minimum shear stresses are larger on the leading edge, while the maximums show only a modest preference. The absolute maximum and minimum values during the 50 revolutions show unique structures that pass over these surfaces, but these will be random and occur multiple times over many hours of operation. These extrema also do not appear in the averages, reinforcing the hypothesis that they are short lived overall.

#### 3.1.2. Pressure

**Figure 9:**
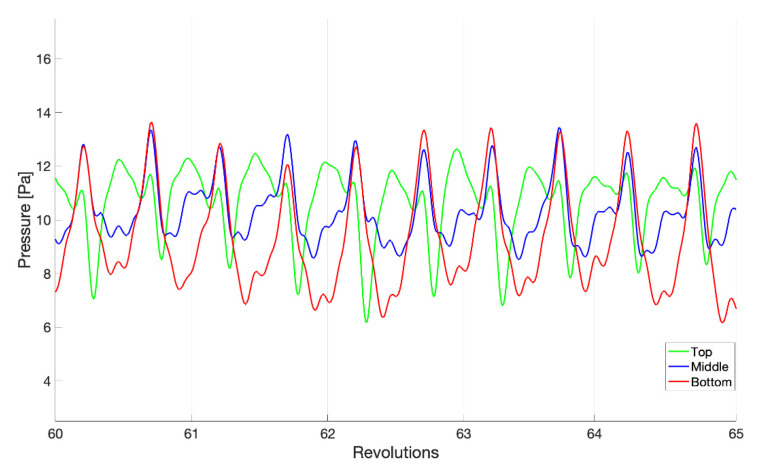
The average pressure for each coupon row for revolutions 60–65. The pressure was adjusted by approximately removing the hydrostatic contribution, leaving the static and dynamic pressures caused by the motion of the baffle.

Different than the shear stress, which is always tangential to a surface, the pressure (or normal stress) that the fluid applies to a surface will also contribute to the loading experienced by a biofilm. As it can be seen in Figure 9, there is a negligible difference in the adjusted gage pressure between the three coupon rows. That said, the twice-per-revolution influence of the baffle has a much more pronounced impact on the loading of the coupons. Similar higher- and lower-frequency responses are still observed, though considering the entire time history, the null hypothesis is rejected below the 1% significance level in all cases. An approximately 65° lag in the peak pressure occurs behind the baffle passage, such that when considering only these time-steps for the bottom and middle rows of coupons, *p* = 0.387. The top coupons do appear to experience a different pressure profile than the middle and bottom coupons, which is likely a result of the proximity to the free surface, the dynamics of which are subject to the liquid building up around the rod structures similar to water in a creek moving around rocks near the surface. This leads to a significant note as to the assumption of how the hydrostatic pressure was removed. The hydrostatic pressure depends on the density of the fluid and the depth of measurement, and as noted above, the depth is approximated as the average of the entire free surface. A 10 Pa shift in the results can occur with a depth variation of 1 mm. That said, a 2–3 Pa amplitude change is observed, indicating that the pressure is still a significantly larger load on the coupon surfaces. However, it is unclear how much of the pressure cycle is pushing into the coupon (positive) or pulling material away from the surfaces (negative) as a result of the hydrostatic approximation.

### 3.2. Experimental Comparison

**Figure 10:**
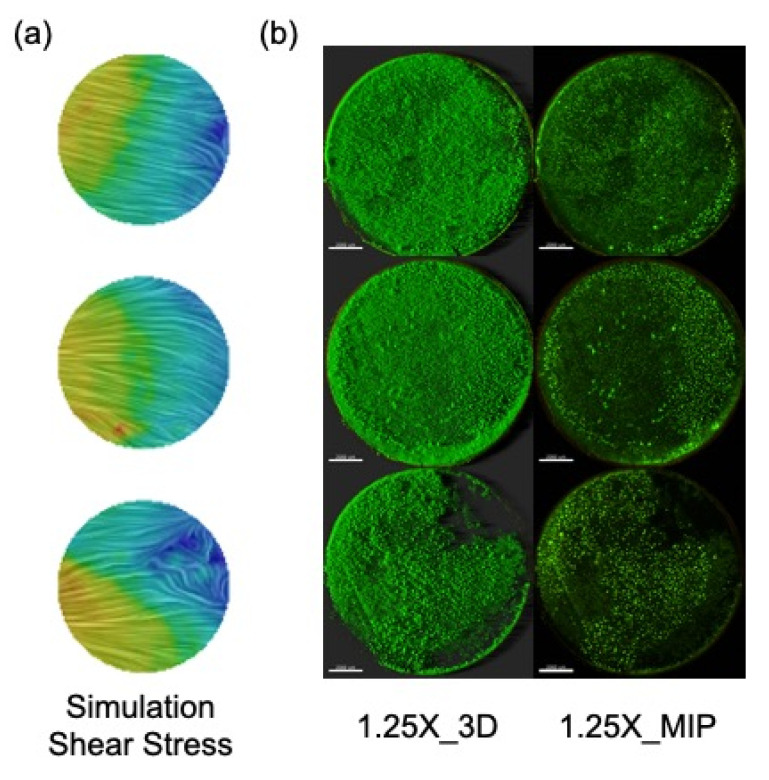
A visual comparison of the (**a**) instantaneous wall shear stress in the simulated reactor and (**b**) lab-grown Pseudomonas aeruginosa. Images were collected using an upright Leica TCS-SP5 Confocal Scanning Laser Microscope and an extra-long 1.25× water immersion objective. The three-dimensional blend images are a three-dimensional representation, including shadowing. The maximum intensity projection (MIP) images show the maximum intensity for all layers of the z-stack. Biofilm grown by Kelli Buckingham-Meyer and Lindsey Miller. Imaging by Lindsey Miller.

The goal of the current research was to use CFD modeling to quantify the fluid dynamics present in the CDC biofilm reactor to understand why certain biofilms grown within this reactor demonstrate high reproducibility. To help visualize the influence the fluid dynamics have on biofilm growth, a *Pseudomonas aeruginosa* biofilm was grown in the CDC biofilm reactor on glass coupons according to ASTM Method E3161 at 125 RPM. The experimental imaging, shown in Figure 10, demonstrates a robust biofilm that is typical of the biofilm that grows in this reactor under this standard. While the side of the coupon that faced the baffle and the location within the rod (top, middle, bottom) were recorded, the orientation of the coupon within the rod was not. Any similarity between the region of flow separation on the bottom coupon and the imaged biofilm is purely coincidental.

## 4. Discussion

Our model demonstrates that the fluid in the CDC biofilm reactor was well mixed with no pockets of stagnation, providing evidence as to why the biofilm grown in this reactor is so reproducible [19]. There is no significant difference in the average shear environment seen between coupons at different depths, and this is confirmed in the numerous studies of biofilms grown within this reactor. A shear bias appears to exist across all coupon surfaces in the rotation direction, with the wall shear stress decreasing in the direction of the baffle motion. In this, the top coupons do demonstrate a slightly narrower distribution of stresses across their surfaces than that seen in the middle and bottom rows; however, the standard deviations of shear across all coupon surfaces are comparable, suggesting the variability in the shear stress is possibly more important than small changes in the average shear stress. Additionally, even though there are small standard deviations in the calculated shear stress across the top, middle, and bottom coupons, the magnitude of the minimum and maximum values suggests no part of the coupon surface is without constantly changing shear. The flows separating around the top and bottom edges of the baffle are suspected to be the significant drivers of the vertical motions seen. Over time, this may contribute to the 3D architecture typical of the biofilm grown in this system.

Pressure at this magnitude has not been suggested as a significant contributor to biofilm growth. That said, the repeated compression and release into and out of a coupon surface may have an influence on the organization of a biofilm and preferential directions of strength. Additionally, while there is some uncertainty in the magnitude of the pressure values observed, the focus should be on the amplitude of the pressure cycles as they are an order magnitude larger than the variations seen in the shearing stresses. There is an observable difference in the pressure profile of the top row coupons, which is likely caused by fluctuations in the height of the free surface.

Through these simulations, the significance of the stir plate magnet on the wall shear stress and pressure is unclear. As noted above, the bottom row of coupons has the smallest *p*-value, indicating potentially more variations over the 50 revolutions analyzed. While the sharp edges of the top and bottom of the baffle would normally be considered to introduce the largest amount of flow separation and mixing within the reactor, the stir plate magnet extends 83% towards the coupons and comprises a little under 20% of the height of the baffle and should not be neglected as a contributing factor. A subsequent simulation without the stir plate magnet would need to be conducted in order to determine the significance of its impact.

To further help conceptualize the magnitude of the shear stresses observed, it may be helpful to provide a rough estimate of an equivalent system that would yield a similar wall shear stress (0.365 Pa). An exact relationship between the wall shear stress and average velocity of water in a smooth, 5 cm diameter pipe exists as the Hagen–Poiseuille equation for laminar flow in a circular pipe. With a laminar assumption, the equation yields an average velocity of 1.28 m/s, or a non-dimensional Reynolds number (Re) of 71,839. As the transition to turbulence within a pipe begins at Re = 2100, this shear stress is only achievable under turbulent conditions for a smooth pipe of this diameter. Additionally, since the wall shear rate is much larger in turbulent flows than that seen in laminar flows, the average velocity required to yield the same wall shear stress would necessarily be lower. Using an iterative approach for turbulent flow, a 5 cm pipe would have a similar wall shear stress to the CDC biofilm reactor coupons if water was flowing between 0.3 and 0.4 m/s. Yet, a uni-directional pipe flow with a constant velocity, such as this, would be unable to produce the range of minimum and maximum shearing stresses observed in the reactor. The CDC biofilm reactor at 125 RPM creates a wholly unique flow and shear environment given its simple motion.

A note needs to be made at this point that highlights a deficiency in the present work and observed more broadly within the field. To the authors’ knowledge, no experimental measurements exist that catalog the velocities, pressures, or shear stresses within biofilm reactors. This will be necessary for any modeling efforts to be validated. Every effort was made in the presented work to use best practices and ensure that the model accuracy was high, but without experimental data to compare these results against, some additional error may exist. Additionally, the coupon orientation and placement in any reactor should be considered when analyzing the results to ensure no flow biases exist. That said, even without a suitably validated simulation, this model clearly demonstrates that the CDC biofilm reactor is a highly mixed system that does not exhibit a simple rotational flow.

CFD is an expansive tool that is able to capture the nuances of the fluid dynamics present in a complex system at a high resolution that would empirically be challenging to obtain due to limitations in the available technology and techniques. This information can be used to understand observable changes in the biofilm growth. Additionally, CFD provides a process for rapidly evaluating changes to a design or the operational conditions and how these influence the fluid dynamics present within a reactor.

Future research will compare simulated results against coupons of biofilms cataloged by location and orientation. Multiple rotation speeds within the CDC biofilm reactor will be considered to understand how flow structures change and whether unique regimes exist. The coupons modeled here are perfectly smooth; however, as biofilms grow or different coupon materials are used, the surface roughness should be considered for its impact on the wall shear stress.

The CDC reactor is demonstrated to be a well-mixed system. This is due to a range of fluid structures, which vary in their size and temporal stability, that are created with the baffle motion. While the system exhibits extrema, the variability in the average shearing stresses across all coupons is minimal and not differentiated between their vertical location. Computational fluid dynamics is demonstrated to be a useful tool in investigating systems generating biofilms and can provide important insights into why a biofilm grows the way it does.

## Figures and Tables

**Table 1:** Row averages of the average, maximum, and minimum total shear stresses for revolutions 20–70.

Row Location	Top	Middle	Bottom
Maximum (Pa)	0.794 ± 0.296	0.784 ± 0.264	0.756 ± 0.201
Average (Pa)	0.358 ± 0.076	0.368 ± 0.063	0.368 ± 0.082
Minimum (Pa)	0.030 ± 0.032	0.030 ± 0.029	0.033 ± 0.035

## Data Availability

Not applicable.
